# A directed genome evolution method to enhance hydrogen production in *Rhodobacter capsulatus*

**DOI:** 10.3389/fmicb.2022.991123

**Published:** 2022-08-24

**Authors:** Emma Barahona, Elisa San Isidro, Laura Sierra-Heras, Inés Álvarez-Melcón, Emilio Jiménez-Vicente, José María Buesa, Juan Imperial, Luis M. Rubio

**Affiliations:** ^1^Centro de Biotecnología y Genómica de Plantas, Universidad Politécnica de Madrid (UPM), Instituto Nacional de Investigación y Tecnología Agraria y Alimentaria (INIA-CSIC), Madrid, Spain; ^2^Departamento de Biotecnología-Biología Vegetal, Escuela Técnica Superior de Ingeniería Agronómica, Alimentaria y de Biosistemas, Universidad Politécnica de Madrid, Madrid, Spain

**Keywords:** nitrogenase, flow cytometry, hydrogenase, biological hydrogen production, *hupA*, mutagenesis

## Abstract

Nitrogenase-dependent H_2_ production by photosynthetic bacteria, such as *Rhodobacter capsulatus*, has been extensively investigated. An important limitation to increase H_2_ production using genetic manipulation is the scarcity of high-throughput screening methods to detect possible overproducing mutants. Previously, we engineered *R. capsulatus* strains that emitted fluorescence in response to H_2_ and used them to identify mutations in the nitrogenase Fe protein leading to H_2_ overproduction. Here, we used ultraviolet light to induce random mutations in the genome of the engineered H_2_-sensing strain, and fluorescent-activated cell sorting to detect and isolate the H_2_-overproducing cells from libraries containing 5 × 10^5^ mutants. Three rounds of mutagenesis and strain selection gradually increased H_2_ production up to 3-fold. The whole genomes of five H_2_ overproducing strains were sequenced and compared to that of the parental sensor strain to determine the basis for H_2_ overproduction. No mutations were present in well-characterized functions related to nitrogen fixation, except for the transcriptional activator *nifA2*. However, several mutations mapped to energy-generating systems and to carbon metabolism-related functions, which could feed reducing power or ATP to nitrogenase. Time-course experiments of nitrogenase depression in batch cultures exposed mismatches between nitrogenase protein levels and their H_2_ and ethylene production activities that suggested energy limitation. Consistently, cultivating in a chemostat produced up to 19-fold more H_2_ than the corresponding batch cultures, revealing the potential of selected H_2_ overproducing strains.

## Introduction

The nitrogen-fixing and photosynthetic purple non-sulfur bacterium (PNSB) *Rhodobacter capsulatus* evolves H_2_ using two genetically distinct nitrogenases, a Mo-nitrogenase and an Fe-only nitrogenase, when cultured under anaerobic, illuminated conditions with organic compounds and in the total absence of nitrogen or in the presence of a poor nitrogen source (Scolnik and Haselkorn, 1984; Strnad et al., 2010). Nitrogenases are two-component enzymes (Bulen and LeComte, 1966) formed by a dinitrogenase (called MoFe protein in the Mo-nitrogenase and FeFe protein in the Fe-only nitrogenase) and a dinitrogenase reductase (called the Fe protein), which catalyze the reduction of N_2_ into NH_3_ in a reaction that also produces, at a minimum, one mol of H_2_ per mol of reduced N_2_ (Seefeldt et al., 2020).

N_2_ + 8 H^+^ + 8 e^−^ + 16 MgATP + 16 H_2_O → H_2_ + 2 NH_3_ + 16 MgADP + 16 P_i_.

The specific substrate-reducing activities of the *R. capsulatus* nitrogenases have been described (Schneider et al., 1991, 1997). The relative H_2_ to NH_3_ production varies with the component ratio and nitrogenase type, being much higher in lower electron fluxes and for the Fe-only nitrogenase compared to the Mo-nitrogenase, making potential H_2_ production much higher than predicted from the above equation.

*Rhodobacter capsulatus* also expresses two hydrogenases: a cytosolic H_2_-sensing [Ni-Fe] hydrogenase encoded by *hupU* and *hupV* (Vignais and Billoud, 2007), and a membrane-bound uptake [Ni–Fe] hydrogenase that catalyzes the reversible reaction 2H^+^ + 2e^−^ ↔ H_2_ (Colbeau et al., 1993; Vignais et al., 2005). This latter enzyme is a heterodimer of the *hupA* and *hupB* gene products. The *hupC* gene product is a cytochrome b-type protein that anchors HupAB to the membrane and receives electrons from HupA (Vignais and Billoud, 2007). Transcription of *hupABC* is controlled by a promoter in response to H_2_ and involves a HupUV H_2_-sensor and a two-component regulatory system consisting of a histidine kinase HupT and a response regulator HupR. In the absence of H_2_, HupT and HupUV interact to form a complex in which HupT has increased auto kinase activity. Autophosphorylated HupT transfers a phosphate group to HupR, which in this state is unable to activate transcription. In the presence of H_2_, HupUV binds H_2_ and HupT is released. Although trans-phosphorylation between HupT and HupR can occur, in this state HupT appears to function rather in a phosphatase mode, leaving HupR in the active, unphosphorylated state, which can now activate transcription (Vignais et al., 2005).

Diverse approaches have been used to increase H_2_ production by genetic manipulation of microorganisms. Increase of H_2_ production by hydrogenase obtained by directed mutagenesis or by removing genes related to their synthesis, regulation or assembly has been reported (Jahn et al., 1994; Masukawa et al., 2002; Liu et al., 2010). Nitrogenases are also excellent H_2_-producing enzymes, and H_2_ production *via* nitrogenase has also been improved, mainly in cyanobacteria (Bandyopadhyay et al., 2010; Masukawa et al., 2010; Skizim et al., 2012) and PNSB (Rey et al., 2007; Kim et al., 2008; Liu et al., 2010; Wang et al., 2010; Barahona et al., 2016; Zhang et al., 2016). An important limitation to improve H_2_-producing enzymes is the scarcity of high-throughput screening methods for H_2_ overproducing microorganisms. In this context, a combination of fluorescence-activated cell sorting (FACS), an engineered *R. capsulatus* strain that generates fluorescein in response to H_2_, and the *in vivo* expression of random variants of the nitrogenase NifH protein in the engineered strain, has been used previously to detect and separate H_2_ overproducing cells in screenings involving over a million variants per experiment (Barahona et al., 2016).

Here, we subjected *R. capsulatus* to directed genome evolution to increase H_2_ production. Random UV mutagenesis of engineered H_2_-sensing strains was combined with FACS to detect and isolate the resulting H_2_-overproducing cells. Three rounds of mutagenesis/selection were performed to achieve 2- to 3-fold increase in H_2_ production over the parental H_2_-sensing strain. The effect of removing H_2_ from the gas phase of *R. capsulatus* cultures to accelerate H_2_ evolution was also investigated.

## Materials and methods

### Bacterial strains, growth media, and growth conditions

Bacterial strains used in this study are listed in Supplementary Table S1. *R. capsulatus* strains were cultivated either in rich YPS medium or RCV minimal medium (Weaver et al., 1975). RCV medium contained 30 mM DL-malate and 10 mM (NH_4_)_2_SO_4_ as sole carbon and nitrogen source, respectively. When required for nitrogenase derepression, ammonium was omitted (RCV_0_) and, when indicated, RCV was supplemented with 10 mM L-serine (in this work, called RCVS). For Petri dishes medium was solidified with 1.5% agar. The medium was supplemented with kanamycin (Km; 50 μg/ml) or rifampicin (Rif; 25 μg/ml) when required. Cultures were incubated at 30°C either under chemotrophic (aerobic) or phototrophic (anaerobic) conditions. Petri dishes were incubated at 30°C inside illuminated (300 lux) anaerobic jars in the presence of anaerobic gas generator bags (AnaeroGen^™^, Thermo Scientific, United States).

To determine growth curves of *R. capsulatus*-derived strains, precultures in RCV medium were diluted to an OD_600_ of 0.15 and transferred in triplicate to 24-multiwell plates. Cultures were grown at 30°C in presence of oxygen with shaking (700 rpm) using a SPECTROstar Nano instrument (BMG LABTECH, Germany) to determine the OD_600,_ and growth was recorded every hour for 44 h.

To derepress nitrogenase in batch cultures, *R. capsulatus* cells precultured in RCV were transferred to 100-ml capped vials containing 60 ml of RCV_0_ medium and adjusted to an initial OD_600_ of 0.18. Vials were sparged with N_2_ to completely remove air. Cultures were grown at 30°C phototrophically (six 60 W light bulbs providing 300 lux at 25 cm of the vials) for 22 h.

Nitrogenase was also derepressed in continuous cultures. The system consisted of an illuminated 300 ml bioreactor containing 150 ml of RCV_0_. The bioreactor was inoculated with anaerobically grown *R. capsulatus* cells to an initial OD_600_ of 0.3 and the reactor was made anaerobic by sparging N_2_ for 1 h (*t* = 0 h). After 16 h of diazotrophic growth, the gas phase was renewed by sparging N_2_ for 1 h. Then, a peristaltic pump was used to replace fresh medium for reactor contents at a flow of 2.5 ml/min until the end of the experiment. At *t* = 39.5 h, the bioreactor was opened again both to release any H_2_ produced and to renew the N_2_ atmosphere by sparging. H_2_ measurements were taken at 0, 16, 17, 22.5, 25.5, 39.5, 40, 46, 49, and 63 h.

### Ultraviolet light mutagenesis

Ultraviolet light (UV light) mutagenesis was performed on *R. capsulatus* wild-type, S1, and S2 strains to generate random mutations along the genome. Each strain was cultured for 48 h on two Petri dishes containing solid YPS medium. One of the two Petri dishes was exposed to UV light while the second plate was used as control. The procedure was carried out in the dark to prevent photoreactivation. After UV light exposure, a loop of cells from each Petri dish was resuspended in 1 ml YPS and vortexed vigorously. Serial dilutions (from 10^−1^ to 10^−6^) were plated onto solid YPS to give 30–300 colonies/plate and incubated at 30°C for 48 h. Colony counts were performed to estimate survival rates. Nine UV treatment times were tested. At 30 s, and at 1, 3, 5, 6, and 8 min, there were no survival differences between UV-treated plates and non-treated controls. At 10 min, the survival rate in UV-treated plates was 90% of the control. At 13 min, the survival rate was 8% for *R. capsulatus* wild type and 10% for the S2 strain. No survivors were obtained after 15 min of UV illumination. Therefore, a 13-min exposure to UV light was used to generate libraries of random mutants. Each library contained *ca.* 3 × 10^9^ colony-forming units.

### Flow cytometry

*Rhodobacter capsulatus* cells grown under diazotrophic conditions in 100-ml capped vials, containing 60 ml of RCVS medium, were collected by centrifugation in Falcon tubes for 15 min at 4°C, 4,500×*g*, resuspended in 5 ml PBS supplemented with 10% glycerol, and incubated for 30 min at 4°C. Cells were then collected, resuspended in 1 ml of an 8:1:1 mixture of PBS, fluorescein di-β-D-galactopyranoside (FDG) and propidium iodide (PI), and incubated at 37°C for 30 min to facilitate FDG entrance into the cells. When cleaved by β-galactosidase, FDG releases fluorescein, which cannot diffuse across the cytoplasmic membrane (Plovins et al., 1994). Cells were collected by centrifugation, resuspended in RCV medium, and analyzed in a FACSVantage (sorter) flow cytometer using an argon ion laser to excite the fluorochrome (488 nm) and a 0.5 μm filament filter to separate very small particles. About 5 × 10^5^ cells were analyzed in each experiment. The cell sorter was programmed with very stringent parameters to separate individual cells leading to clonal populations. Only a few cells from a subpopulation exhibiting 10–100-fold more fluorescence than the population average were sorted and recovered in 96-well microplates containing RCV medium. These stringent sorting conditions were chosen to ensure that just one highly fluorescent cell, or none, went into the receiving well. As a result, only a single microplate was filled in a 2-h experiment, and about one-half of the wells produced no growth when their contents were used as inoculum. Grown cultures were then diluted to normalize their OD and transferred to RCVS medium in 96-well microplates to derepress nitrogenase overnight and prepare for 4-methylumbelliferone β-D-galactopyranoside (MUG) assays.

### 4-Methylumbelliferone β-D-galactopyranoside activity assays

4-Methylumbelliferone β-D-galactopyranoside β-galactosidase activity assays were carried out as described in (Barahona et al., 2016). *R. capsulatus* cultures were incubated overnight under diazotrophic conditions inside a glove box in a 96-well plate (black/clear Optilux^™^ flat bottom; BD Biosciences) covered with a transparent adhesive sealer. Portions of 120 μl from each culture were transferred to a 96-well microplate containing 100 μl of Z-Buffer (Miller, 1972) in each well, then supplemented with 25 μl MUG solution (1 mg/ml solution in dimethyl sulfoxide) and incubated at room temperature for 2 h in darkness. MUG hydrolysis by β-galactosidase was quantified by fluorescence emission at 445 nm (372 nm excitation wavelength) in a Genios Pro (Tecan) microplate fluorometer.

### *In vivo* nitrogenase acetylene reduction assay

To determine acetylene reduction activity in *R. capsulatus*, 1 ml portions of cultures grown under diazotrophic conditions were transferred to 9-ml sealed vials with a 94% N_2_/6% acetylene gas phase and incubated at 30°C in presence of light for 30 min. Ethylene formation was detected in 50 μl samples withdrawn from the gas phase by using a Shimadzu GC-2014 gas chromatograph equipped with a 9-ft long, 1/8-in diameter Porapak R column. *In vivo* nitrogenase activity units are defined as nmol ethylene formed per min per ml of culture at an OD_600_ equal to 1 (nmol C_2_H_4_ min^−1^ OD_600_^−1^).

### H_2_ production measurements

To determine H_2_ production in *R. capsulatus* cultures grown under diazotrophic conditions, 250 μl samples were withdrawn at the indicated times from the gas phase of 100 ml capped vials. H_2_ production in *R. capsulatus* continuous cultures was also determined in 250 μl gas phase samples withdrawn at the indicated times. Samples were injected in a Shimadzu GC-8A gas chromatograph equipped with a 6-ft long, 1/8-in diameter Molecular Sieve column 5A. Each measurement had two technical replicates per biological replicate. H_2_ production activity is presented either as total H_2_ released or as H_2_ released per hour and ml of culture (OD_600_ = 1).

### Immunoblot detection of NifH and NifDK proteins

For SDS-PAGE, cells from 1-ml culture samples were collected by centrifugation, resuspended in 2× Laemmli sample buffer supplemented with 0.1 M dithiothreitol (to a concentration equivalent to an OD_600_ of 4), and electrophoresed in 12% acrylamide/bisacrylamide (29:1) gels. For immunoblot analysis, proteins were transferred to nitrocellulose membranes for 40 min at 20 V using a Transfer-Blot® Semi Dry system (Bio-Rad). Immunoblot analyses were carried out with antibodies raised against a 1:1 mixture of *Azotobacter vinelandii* and *Rhodospirillum rubrum* NifH proteins (1:2,500 dilution) or with antibodies raised against *R. capsulatus* NifDK (1:2,000 dilution; antibody kindly donated by Yves Jouanneau, CNRS, Grenoble). Secondary HRP-conjugated anti-rabbit antibody (Invitrogen, United States) was used at 1:15,000 dilution.

### Whole-genome DNA sequencing and data analysis

For second-generation genome sequencing, total DNA from bacterial cultures was isolated using the NZY Tissue gDNA isolation kit (NZYTech), following the manufacturer’s instructions, and eluted in a final volume of 100 μl. A negative control that contained no sample was included in the DNA isolation process to check for cross-contamination during the experiments. DNA samples were quantified using the Qubit dsDNA HS Assay kit (Thermo Fisher Scientific) and sequenced by an external service provider (AllGenetics & Biology SL). Briefly, libraries were constructed using the Nextera XT DNA Library Prep kit (Illumina) according to the manufacturer’s instructions, and they were dual indexed for post-sequencing demultiplexing. The fragment size distribution of the libraries was checked with an Agilent 2,100 Bioanalyzer using the Agilent DNA 1000 Kit. Libraries were quantified with the Qubit dsDNA HS Assay Kit and pooled in equimolar amounts. Pooled libraries were then sequenced with an Illumina MiSeq (2 × PE, 300 bp) sequencer.

For third-generation genome sequencing, total DNA extraction from S2– *R. capsulatus* culture was performed using DNeasy Blood & Tissue Kit (QIAGEN) according to PacBio guidelines for handling high-molecular-weight DNA for successful constructions of SMRTbell^™^ libraries. Quick Ampure XP bead clean-up was then performed to remove RNA contamination. DNA quality (i.e., integrity, purity, and concentration) necessary for library preparations was evaluated from gel images of DNA samples and by Qubit^®^ fluorimetry. Barcoded libraries were prepared, and the size was selected by performing one 0.45 × (sample volume to beads volume ratio) clean followed by another 0.4 x clean using Ampure beads to remove DNA fragments smaller than 3–5 kb. Sequence data were demultiplexed and genomes assembled using PacBio’s Microbial Assembly Tool.

Raw genome sequencing reads were processed to correct or eliminate erroneous reads. Error correction algorithms, from simple trimming processes using base quality scores to complex error correction approaches based on the frequency of erroneous reads in the set being assembled, were carried out. Subsequently, single-nucleotide polymorphisms (SNV) were mapped to the S2– *R. capsulatus* genome used as reference. Initial DNA sequence comparisons detected more than a thousand differences between the reference genome and UV-mutated derivatives, but most of those laid in regions of lower sequence coverage and were considered population polymorphisms. Thus, only SNVs present in more than 65% of the reads, and that were also present in subsequent strain derivatives (3 rounds of mutagenesis were performed), were considered for further analysis. All procedures were performed using Geneious version 6.1.8 software. SNVs were confirmed by performing Basic Local Alignment Search Tool (BLAST) against the reference genome.

### Statistical analysis

Statistical analyses were carried out using Prism software. One-way ANOVA tests were performed to compare the means of multiple sets of data (*p* < 0.05). Adjusted *p* values were determined by the Bonferroni test.

## Results

We sought to develop a method to generate and *in vivo* identify H_2_ overproducing PNSB variants that could be easily implemented in the set-up of an academic R&D laboratory. This method starts by generating a huge library of random mutants that is subsequently screened in groups of half a million at a time by FACS using a fluorescence signal as proxy for H_2_ production. The selection procedure favors speed and standardization, and balances, on one hand, a significant increase in fluorescence signal with, on the other hand, obtaining a manageable number of selected overproducing strains. We found out that about 1% of the mutant population emitted 10-100-fold higher signal than average, and that a 2–3-h FACS experiment could sort out and collect 96 of them in a single plate. Because the number of isolated clones was relatively small, a growth test and secondary H_2_ production proxy screening were then performed to validate the FACS selection before real H_2_ production was determined. To “fix” advantageous mutations for H_2_ production, we decided to pursue stepwise increments rather than performing a deeper– and much longer–screening of an individual mutagenic library. Thus, the 2–3 highest H_2_ overproducing mutants were used in subsequent cycles of mutagenesis and screening. At the end, a collection of H_2_ overproducing *R. capsulatus* mutants was analyzed by whole genome sequencing to gain insights into the genetic basis of their phenotypes and to establish the mutation genealogy.

### First round of UV mutagenesis and screening for H_2_-overproducing strains

**Figure 1:**
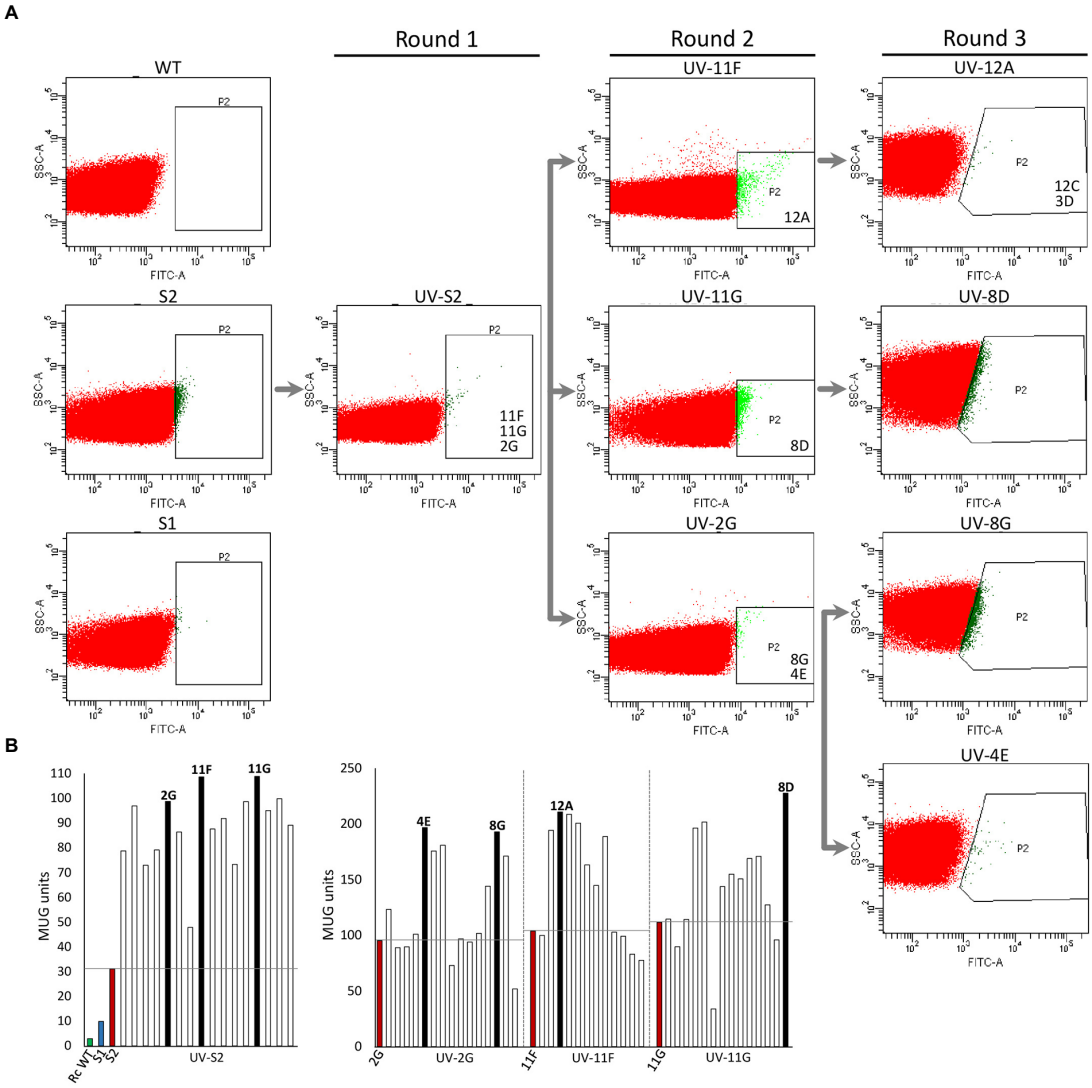
High-throughput selection of *Rhodobacter capsulatus* cells emitting fluorescence in response to H_2_. **(A)** Fluorescence activated cell sorting. Dot-plot showing side-scattered light (SSC) versus fluorescence generated by fluorescein isothiocyanate (FITC) in *R. capsulatus* populations of wild type, S1, S2, UV-S2 (pool of S2 cells after UV treatment), and derivative strains obtained by further rounds of UV mutagenesis. P2 indicates areas used for cell sorting into 96-well plates. Strains analyzed in depth in this study (*e. g.* 11F, 11G, and 2G) are shown in the P2 area from which they were isolated. **(B)** Examples of beta-galactosidase activity (MUG hydrolysis in 96-well plate format) of clones sorted by FACS. Left, UV-S2. Right, UV-2G, UV-11F, and UV-11G sorted populations. Green and blue bars show wild-type and S1 and S2 activities, respectively. Red bars show activities of strain subject to each mutagenic treatment. Black bars represent activities of clones selected for further rounds of mutagenesis.

**Figure 2:**
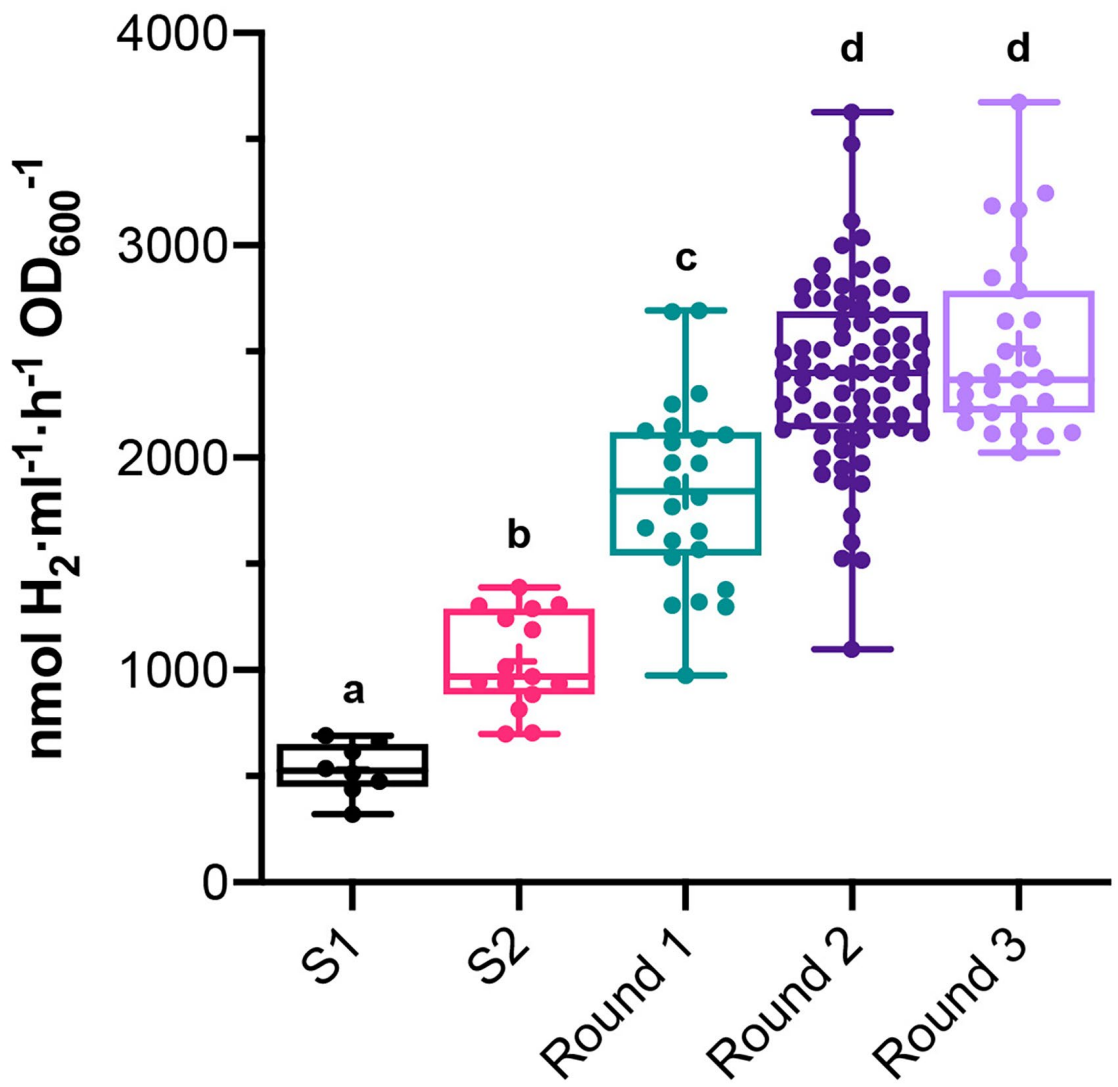
*In vivo* H_2_ production in batch cultures of clones that had high β-galactosidase activity in previous MUG assays. Measurements were taken after each round of UV mutagenesis. *In vivo* H_2_ production of S1 and S2 are shown as reference. Boxes show 25th to 75th percentiles, the median (line), and the mean (+). Whiskers show from minimum to maximum values. Each dot represents H_2_ production of a different clone. Different letters indicate statistically significant differences (*p* < 0.05 for S1 vs. S2, and *p* < 0.0001 for the rest of group comparisons).

**Figure 3:**
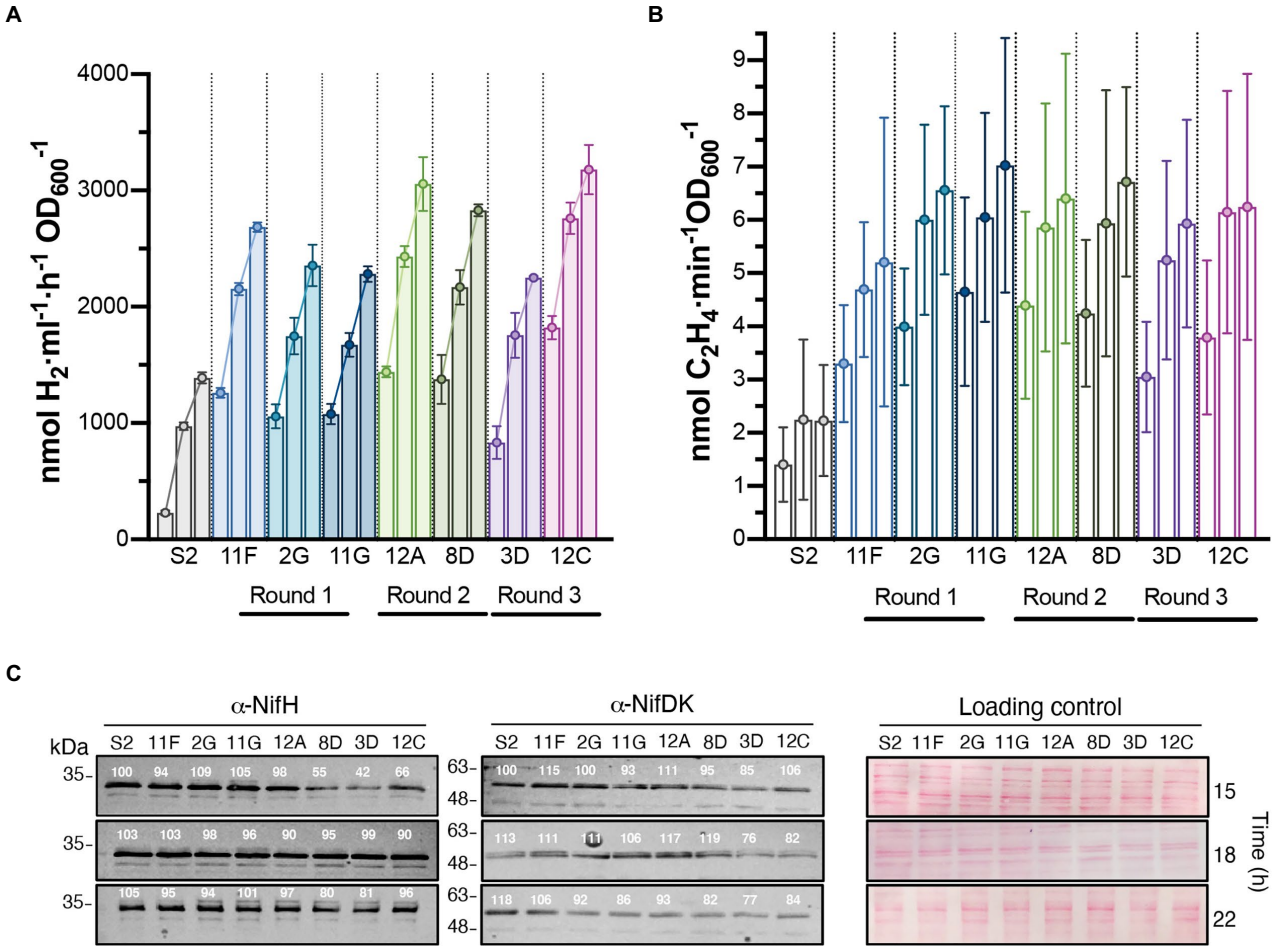
Time course of H_2_ production in batch cultures of selected strains isolated after each round of UV mutagenesis. *In vivo* H_2_ **(A)** and ethylene **(B)** production of S2 and the highest H_2_ overproducing variants. The three bars of each strain represent activities at 15, 18, and 22 h after the start of nitrogenase depression. Data represent the mean ± SD of biological replicates (*n* = 6 for H_2_, *n* = 4 for ethylene). Each biological replicate had 2 technical replicates. Statistically significant differences in H_2_ production between S2 and H_2_ overproducers existed at all measured times (*p* < 0.0001). Differences in ethylene production between S2 and H_2_ overproducers were statistically significant at 22 h (*p* < 0.05). **(C)** Immunoblot detection of nitrogenase NifH and NifDK components in cell-free soluble extracts from cultures shown in panel B. Normalized quantification of band intensity is indicated above each band. Ponceau staining of membranes is shown as loading control. Full uncropped gels are available as Supplementary Figure S5.

*Rhodobacter capsulatus* S1 and S2 sensor strains contain a copy of *lacZ* (P*hupA::lacZ*) integrated in the chromosome between *hypF* and *hupA* (Supplementary Table S1). They express LacZ and catalyze the formation of fluorescein from fluorescein di-β-D-galactopyranoside (FDG) in response to endogenously produced H_2_ (Barahona et al., 2016). In addition, S2 lacks the *hupAB* structural genes for the uptake-hydrogenase and is unable to consume H_2_. Random genome-wide mutagenesis of S2 was performed as starting point to generate strains with enhanced *in vivo* H_2_ production. S2 cells growing on solid YPS medium were exposed to UV light, serially diluted, and plated again to assess survival. About 3 × 10^9^ colony-forming units (10% survival rate) were recovered after mutagenesis. Mutagenized cells (UV-S2) were pooled, derepressed for nitrogenase in RCVS medium, incubated with FDG, and analyzed by FACS flow cytometry (Figure 1A). Wild-type, S1, and S2 cells derepressed for nitrogenase and treated with FDG were used as controls. About 5 × 10^5^ mutagenic events were processed per FACS sample. UV-S2 cells emitting 10 to 100-fold more fluorescence than the main population (1.4% of the total population in the P2 areas of Figure 1A) were sorted by the flow cytometer and some of them were collected into a 96-well microplate containing RCV medium. After incubation, growth was observed in 52% of the inoculated wells. Grown cultures were then transferred to RCVS medium inside an anaerobic glovebox for nitrogenase derepression and secondary MUG-based β-galactosidase activity determinations. Fourteen mutants exhibited at least 2.5-fold higher MUG-derived fluorescence than S2 (Figure 1B). Derepressed cultures showed statistically significant increases in H_2_ production rates compared to S1 and S2 (see Round 1 box in Figure 2). S2-derived strains 2G, 11F, and 11G were selected as the highest H_2_ producers and further analyzed in time-course H_2_-production experiments (Figure 3A).

### Additional rounds of mutagenesis and screening to further increase H_2_ production

2G, 11F, and 11G strains were separately subjected to a second round of UV mutagenesis and FACS screening. The percentage of cells in selective P2 areas of FACS was 0.027, 0.5, and 0.01%, for UV-11F, UV-11G, and UV-2G, respectively (Figure 1A). As in round 1, cultures presenting over twice the β-galactosidase activity of their parental strains were monitored for H_2_ production, and the highest H_2_ producers were selected (Figure 1B). Selected strains were used in subsequent rounds of UV mutagenesis and screening while statistically significant increases in H_2_ production rates were observed. Significant differences were observed between S1, S2, and mutants generated in mutagenic rounds 1 and round 2, but not between round 2 and round 3 mutants (Figure 2). Therefore, no further rounds of mutagenesis were performed after round 3. Supplementary Figure S1 shows the genealogy of *R. capsulatus* strains generated in this work.

The H_2_ production rates of selected strains subject to subsequent rounds of UV mutagenesis are shown in Figure 3A. H_2_ production rates of additional derivative strains can be found in Supplementary Figure S2. Rates were determined in time-course experiments after nitrogenase derepression, and statistically significant differences between S2 and derivative strains were observed at all investigated times. Strains 12A, 8D, and 12C stood out as the highest overproducers, with average production rates of 3,056, 2,830, and 3,180 nmol H_2_ ml^−1^ h^−1^ OD_600_^−1^, respectively (compared to average S2 production of 1,388 nmol H_2_ ml^−1^ h^−1^ OD_600_^−1^).

Mo-nitrogenase is the enzyme responsible for H_2_ production in *R. capsulatus* growing photoheterotrophically in the absence of fixed N and when the growth medium contains Mo. *In vivo* nitrogenase activity in S2 and derivative strains was estimated by the acetylene-to-ethylene reduction method. Ethylene production was lower in S2 than in the derivative strains at all analyzed times, although differences were only statistically significant 22 h after derepression (Figure 3B). Correlations between H_2_ and ethylene production activities were observed for all strains, indicating that H_2_ overproduction phenotypes were not due to changes in nitrogenase substrate specificity (*i. e*. preference of H^+^ over acetylene), as reported previously for *nifH* mutant experiments (Barahona et al., 2016). On the contrary, nitrogenase activities did not correlate with the accumulation of the NifH and NifDK components of Mo-nitrogenase, as S2 accumulated more Nif polypeptides than most of its derivative strains (Figure 3C). 8D, 3D, and 12C had slightly altered NifH and NifDK accumulation profiles compared to the other strains.

### Improving H_2_ production under continuous culture conditions with intermittent gas exchange

After maximum accumulation of nitrogenase polypeptides occurred, the rates of H_2_ production decelerated over time in all strains and, finally, decreased between 38 and 39 h (Supplementary Figure S3). Consistently, acetylene reduction activities were lower at 39 h than at 22 h after nitrogenase derepression (data not shown). The slight decrease of NifH and NifDK accumulation over time (Figure 3C) cannot entirely account for the magnitude of this effect, and therefore, additional factors must be involved. One possibility is the limitation of electrons and ATP supply to nitrogenase. However, because aerobic non-diazotrophic growth also stopped at similar culture OD (Supplementary Figure S4), it appears that the observed decrease was caused by more general, physiological causes. Typically, substrate limitation or waste accumulation are responsible for batch cultures entering the stationary growth phase. Therefore, continuous culture experiments were undertaken.

**Figure 4:**
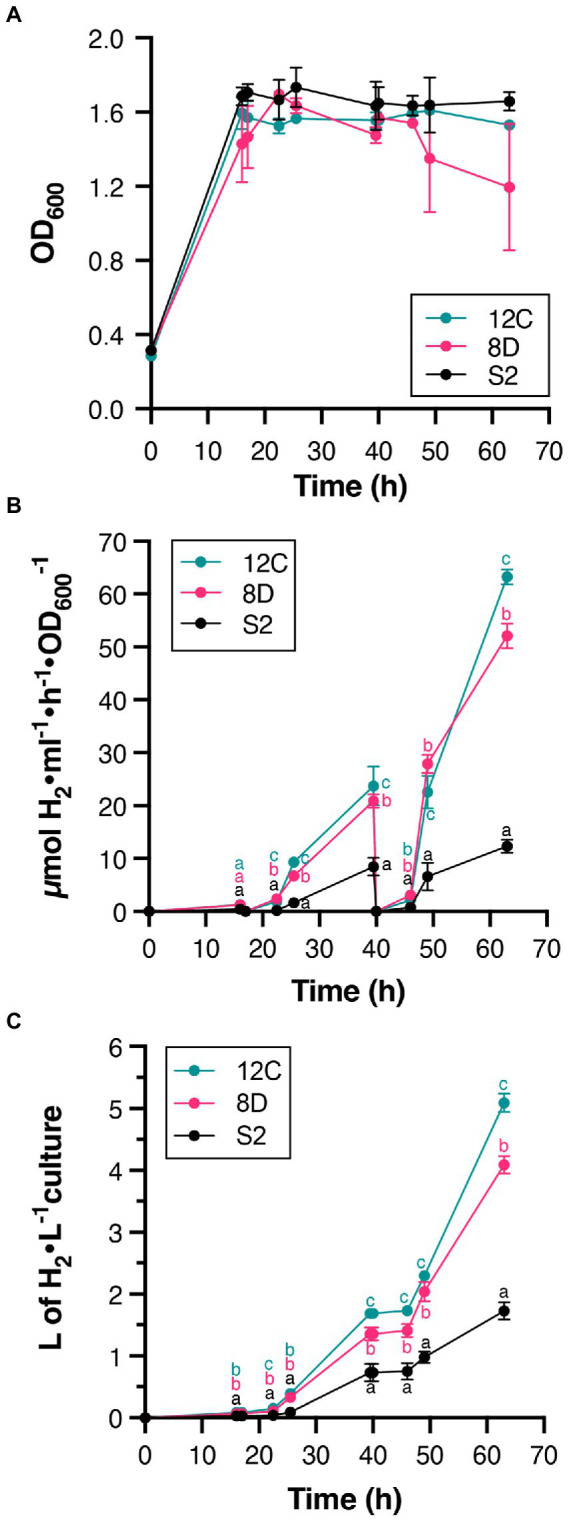
H_2_ production in continuous cultures of S2, 8D, and 12C strains. **(A)** Stability of culture OD during the experiment. Continuous culture conditions were established once the OD_600_ reached 1.6. **(B)** Time course of *in vivo* H_2_ accumulation. Note the disappearance of accumulated H_2_ at times 17 and 40 h due to complete regeneration of the gas phase. **(C).** The total amount of H_2_ produced. Statistically significant differences in H_2_ production between S2 and H_2_ overproducers existed at all measured times. Different letters (a, b, or c) indicate statistically significant differences (*p* < 0.05). In all panels, data represent the mean ± SD of biological replicates (*n* = 4 for S2, *n* = 2 for 8D and 12C) with two technical replicates each.

Continuous culture conditions were established that maintained culture density at OD_600_ = 1.6 (Figure 4A), and H_2_ production by S2, 8D, and 12C strains was determined during 63-h experiments (Figures 4B,C). Periodic atmosphere changes to replenish N_2_ and to remove accumulated H_2_ were also implemented. Figure 4B shows that all accumulated H_2_ disappeared after atmosphere regeneration at 17 and 40 h. H_2_ production rates increased during the experiment and were always higher in 12C and 8D than S2 (Figure 4). At 63 h, strains 12C and 8D produced 63 ± 1 μmol H_2_ h^−1^ ml^−1^ OD_600_^−1^ and 52 ± 2 μmol H_2_ h^−1^ ml^−1^ OD_600_^−1^, respectively, compared to 12 ± 1 μmol H_2_ h^−1^ ml^−1^ OD_600_^−1^ in the S2 strain. Comparison with their maximum H_2_ production rates in batch cultures (*ca.* 1–3 μmol H_2_ h^−1^ ml^−1^ OD_600_^−1^, see Figure 3A) indicated that continuous culture conditions effectively removed some limitations to H_2_ production. The total volume of H_2_ produced in 63 h by strains 8D and 12C was 4 and 5 l H_2_ per L of culture, respectively (Figure 4C).

### Identification of mutations in *Rhodobacter capsulatus* H_2_ overproducing strains

Mutations present in 11F, 12A, 12C, 3D, and 8D strains were identified by whole genome sequencing (Illumina MiSeq, PE 2 × 300 bp, 100 × coverage) and mapped to the *R. capsulatus* S2 chromosomal DNA sequence. Due to the large number of differences in the DNA sequences of the mutants and S2, only SNVs that were present in more than 65% of the mutant reads, and that were also present in subsequent strain derivatives, were considered. These stringent criteria represent a conservative approach. Therefore, the chosen mutations might not encompass all possible changes but rather represent the minimum relevant changes caused by UV that, altogether, affect H_2_ production phenotype. Mutations identified in the selected H_2_ overproducing strains are listed in Table 1 and Supplementary Table S2. SNVs were identified that resulted in amino acid changes within functional gene sequences or changes in promoters and terminator regions. Point mutations leading to nucleotide deletions that create frameshifts were also observed. A total of 35 genomic changes, uniformly and randomly distributed across the genome, were identified among strains (Supplementary Table S2). These changes included two deletions and 31 SNVs, 21 of which resulted in amino acid changes. In seven cases, two mutations were localized very close in the same ORFs. As expected, parental variations were maintained in second- and third-round mutants (except for *eda* mutation in 11F).

**Table 1:** Mutations accumulated in *Rhodobacter capsulatus* H_2_ overproducing strains.

Strain	Accumulated mutations	Relevant genes affected	Proposed function
S2	1 mutation	*hupAB*	Uptake hydrogenase (H_2_ consumption)
11F	S2 + 7 mutations	*rcc02232*	FAD dependent oxidoreductase (Energy production)
12A	11F + 5 mutations	*rcc01477*	FAD-dependent oxidoreductase (Energy production)
		*araB*	Ribulokinase (Carbohydrate metabolism)
8D	S2 + 5 mutations	*ackA2*	Acetate kinase (Carbon metabolism)
3D	12A + 5 mutations	*ndh*	NADH dehydrogenase (Energy production)
12C	12A + 6 mutations	*metH3*	Methionine synthase (SAM biosynthesis)
		*nifA2*	Nif transcriptional regulator (Nitrogen fixation)

Table 1 lists mutations in genes that could be relevant to H_2_-overproducing phenotypes. Deletion of structural uptake hydrogenase genes (*hupAB*) was confirmed in S2 and all derivatives were sequenced in this study. Obviously, the absence of HupAB prevented consumption of H_2_ produced by nitrogenase. 8D had a mutation in *ackA2*, encoding acetate kinase, which possibly affects carbon metabolism. 11F had a mutation in *rcc02232*, a gene linked to a nitrogen fixation gene cluster that encodes a FAD-dependent oxidoreductase, and possibly affects electron donation to nitrogenase. Importantly, this mutation was carried over to 12A, 12C, and 3D derivative strains. 12A had additional mutations in *rcc01477* (encoding another FAD-dependent oxidoreductase) and *araB* (encoding a ribulokinase involved in arabinose catabolism), with implications in carbohydrate metabolism and electron transfer. In addition to mutations carried over from 11F and 12A, 3D has a mutation in a *ndh* gene encoding a subunit of an NADH dehydrogenase, and thus involved in energy production. Most importantly, strain 12C has mutations in *metH3*, encoding a methionine synthase necessary for *S*-adenosylmethionine (SAM) biosynthesis, and in *nifA2*, a second, initially redundant, *nif*-specific transcriptional regulator located in *nif* cluster B (Demtröder et al., 2019). SAM is required for the biosynthesis of the iron-molybdenum cofactor of nitrogenase by NifB (Curatti et al., 2006). Notably, NifH and NifDK accumulation in 12C was not much lower than in the other analyzed strains (Figure 3C).

## Discussion

Biohydrogen generation by photosynthetic bacteria, such as PNSB, exhibits low productivity making them unsuitable to generate H_2_ for large-scale applications (Chandrasekhar et al., 2015). However, their production has been insufficiently explored, being underdeveloped, and remains a promising renewable source of H_2_ considering the energy input (sunlight) and purity of product output (Gupta et al., 2013; Chandrasekhar et al., 2015; Jiménez-Llanos et al., 2020; Chai et al., 2021). Several strategies to enhance photo-fermentative biohydrogen production have been described, such as immobilization of bacteria for continuous H_2_ production (Fiβler et al., 1995; Elkahlout et al., 2019), modification of carbon substrates, nitrogen source, and micronutrients contained in the H_2_ production medium (Liu et al., 2009; Laocharoen and Reungsang, 2014; Chen et al., 2017), and genetic modifications (Barahona et al., 2016; Feng et al., 2018b; Ma et al., 2021), among others.

Previously, we were able to increase H_2_ production by *R. capsulatus* 10-fold through the development of a biotechnological tool for the detection of H_2_-overproducing mutants expressing randomly generated nitrogenase variants (Barahona et al., 2016). The tool was based on the sensing hydrogenase of *R. capsulatus* and produced a fluorescent signal proportional to the amount of H_2_ produced by each variant. Here, we have used it for genome-wide screening of mutations leading to enhanced H_2_ production, thus expanding the impact of this tool.

This work focuses on methodology for non-designed enhancement of H_2_ production activity through the detection of rare overproducers within a very large population of cells. One outcome from this work is that this method did not accumulate mutations on nitrogenase or hydrogenase genes, which would be obvious targets for a designed mutant strategy. It appears that a panoply of mutations, probably resulting in small production increments, underly the H_2_ overproducing phenotype. As a result, the proposed methodology does not allow to unequivocally assign specific genotypes as responsible for the observed H_2_ overproducing phenotype, and the putative contribution of each one of the identified mutations towards this phenotype will require further experimentation.

To identify genes and pathways involved in H_2_ metabolism, we performed random DNA mutagenesis of *R. capsulatus* S2 using UV mutagenesis. Random mutagenesis of bacteria and algae using both physical (UV) and chemical (e.g. ethyl methane sulfonate) mutagens has been used extensively to improve microorganism activities useful at industrial scale (Joshi et al., 2013; Lee et al., 2014; Perin et al., 2015). UV radiation (250–290 nm) induces either formation of thymine dimers that cause transition of G and C to adenine A and thymine T and/or deletion of A–T base pairs in the DNA. *R. capsulatus* has an efficient photoreactivation system that repairs DNA damage induced by UV (Barbe et al., 1987). Thus, in this work, UV mutagenesis was performed in absence of visible light. Point mutations in *R. capsulatus* regulatory sequences and other key genes could result in enhanced H_2_ production. The possibility of mutation bias resulting from the UV treatment cannot be ruled out as no other chemical random mutagenesis was performed in parallel. The survivors of mutagenic treatment were screened in groups of 5 × 10^5^ CFU. About 0.01 to 1% of them exhibited enhanced fluorescence compared to the population average and the parental strain. The effectiveness of FACS high-throughput screening was confirmed by secondary β-galactosidase and H_2_ production activities.

The greatest differences in H_2_ production were observed at 22 h, where all selected strains produced higher levels than S2. The highest H_2_-overproducing strains were subject to subsequent rounds of UV mutagenesis and FACS to continue improving H_2_ production. Thus, accumulation of mutations after each round could be closely linked to the enhancement of H_2_ production. Differences in H_2_ production rates were lower at 39 than 22 h, probably due to depletion of nutrients in batch cultures, as previously reported (Bianchi et al., 2010; Boran et al., 2010; Feng et al., 2018a). Overall, H_2_ production was enhanced 6-fold by deleting the *hupAB* genes (uptake hydrogenase) and by performing random mutagenesis. In addition, continuous cultures of selected mutant strains were shown to produce 19-fold more H_2_ than the corresponding batch cultures.

Nitrogenase variants greatly enhancing H_2_ production have been previously obtained. The most active H_2_ producers lost the ability to reduce acetylene into ethylene (Barahona et al., 2016; Zheng and Harwood, 2019). In contrast to the nitrogenase-focused approach, all variants analyzed by genome-wide screening exhibited higher acetylene reduction activities than the S2 parental strain, although nitrogenase structural proteins, NifH and NifDK, were expressed at similar levels. This observation suggests that changes selected in the genome of variants do not affect nitrogenase substrate specificity and could be altering either (i) other nitrogenase-related proteins, or proteins that are involved in nitrogen fixation-related pathways, or (ii) regulatory regions or factors. One aspect not investigated here was the possible contribution of the Fe-only nitrogenase to the H_2_ overproduction phenotype. A comparative characterization of H_2_ production by Mo- and Fe-only nitrogenases in a *hupB*^−^ mutant of *R. capsulatus* indicated higher production from the Fe-only nitrogenase operating under N_2_ atmosphere (Krahn et al., 1996). However, in our experiments, the Fe-only nitrogenase is repressed because the culture medium contains molybdate. Thus, mutations derepressing its expression in presence of molybdate would be required.

Regarding regulatory mutations, it is noteworthy that round 2 mutant 12C, one of the strongest H_2_ overproducers, contains a mutation in *nifA2. R. capsulatus* contains two copies of the *nifA* transcriptional activator gene, *nifA1* and *nifA2*, and their gene products equally activate both *nif* and *anf* promoters (Demtröder et al., 2019). However, the regulation of *nifA2* differs from that of *nifA1* in that it is subject not only to NtrC nitrogen control, but also to the general, redox-responding RegAB regulatory system (Elsen et al., 2000).

Regarding mutations in nitrogenase-related functions or pathways, none of the mutations accumulated in selected H_2_ overproducers targeted well-characterized functions related to nitrogen fixation. However, some of them affect genes with proposed functions that could be relevant to nitrogen fixation. Strain 11F –and hence 12A and its derivatives– contained a mutation in *rcc02232*, a proposed FAD oxidoreductase, while 12A and its derivatives accumulated an additional mutation in *rcc01477*, another proposed FAD oxidoreductase. FAD-dependent oxidoreductases play a role in energy-generating systems and here they may be part of an ancillary system, unknown until now, that can feed reducing power or ATP to the nitrogenase system. This possibility is further substantiated by the presence of a *ndh* mutation in 3D, 12A-derived strain. Although many NADH oxidases have been linked to oxidative stress control, regeneration of NAD^+^ is also critical for cellular energy generation.

Another set of selected mutations is that of C metabolism-related functions. In strain 12A and derivatives, a mutation in *araB,* a ribulokinase, was selected, while strain 8D showed a mutation in *ackA2*, an acetate kinase. Gene *ackA2* has been implicated in a 1,2-propanediol degradation pathway that is overexpressed in mutants in the RegAB general redox regulatory system (Schindel and Bauer, 2016). AraB, on the other hand, catalyzes the conversion of ribulose to ribulose-5-phosphate (Agarwal et al., 2012). Ribulose-5-phosphate, besides constituting an energy vector, can feed into the pentose phosphate pathway. It has been reported that polysaccharide production substantially increases in *R. capsulatus* mutants lacking nitrogenase activity (Klein et al., 1991). This suggests that nitrogen fixation and polysaccharide production pathways may be competing for metabolites or reducing power. Were this the case, it is possible that a mutation in *araB* could result in higher energy availability to nitrogenase, thus explaining its higher H_2_ production.

Finally, one of the mutations in the overproducing strain 12C is located within the *metH3* gene that codes for a methionine synthase. Methionine is a precursor of S-adenosyl-methionine (SAM), and SAM is required for NifB activity in the synthesis of the NifB-co cofactor, a precursor to the nitrogenase catalytic cofactor, FeMo-co (Buren et al., 2020). However, in the absence of data regarding alteration of SAM levels in strain 12C, the effect of this mutation is difficult to rationalize. This gene could simply be involved in the relative availability of sulfur that might affect the relative NH_3_ vs. H_2_ produced. Or its mutation might alter the relative expression of Mo– vs. Fe– only nitrogenase affecting H_2_ produced.

At present it is difficult to rationalize the role of the above mutations that result in a nitrogenase able to efficiently evolve H_2_ while not being affected in its ability to reduce N_2_, within a specific framework. Further investigation of the phenotypes of these mutations will probably clarify some of the complex metabolic interactions that the proposed functions of the genes implicated suggest. This will probably unearth subtle redox and energy interactions in the nitrogen fixation process that have so far escaped detection but that are, nonetheless, important to determine the fate of electrons through nitrogenase. In this respect, it is important to keep in mind that alterations in the electron flux through nitrogenase can alter its preference for different substrates (*e. g.* protons or N_2_). Purified nitrogenase allocates 25% of the electrons fed to it to proton reduction (Simpson and Burris, 1984), and this has been mechanistically explained (Seefeldt et al., 2020). However, *in vivo*, higher ratios of proton reduction have been observed, especially in symbiotic systems under stressful conditions for the legume symbiont (Schubert and Evans, 1976), an observation that is also commonly explained by the complexities of the nitrogenase catalytic mechanism: given that nitrogenase needs to store six electrons for the stepwise reduction of N_2_ to NH_3_, it is expected that when the electron flux is suboptimal, electrons can leak out to protons and be lost as H_2_. Hence, any mutations that negatively affect the efficiency of the flux of electrons through nitrogenase would probably result in a higher proportion of electrons allocated to protons, and thus a higher production of H_2_. The selected mutations would fit within this category.

It is surprising that no nitrogenase mutations were identified in this screening. Future developments should include combining mutations identified in (Barahona et al., 2016) and this study, as well as exploiting the Fe-only nitrogenase activity by deregulating it or by changing culture conditions to express it.

## Data availability statement

The datasets presented in this study can be found in online repositories. The names of the repository/repositories and accession number(s) can be found at: GenBank BioProject PRJNA859503.

## Author contributions

LR initiated and directed this research. EB, EI, LS-H, and IÁ-M performed molecular biology. EB performed cellular biology, flow cytometry, and biochemical assays. EJ-V contributed biochemical assays. JB and EB performed fermentations. EB and JI performed mutant sequence analysis. EB and LR performed the experimental design and data analysis. EB, JI, and LR wrote the manuscript. All authors contributed to the article and approved the submitted version.

## Funding

European Research Council starting grant 205442 funded the generation of hydrogen sensor strains. Fundación Iberdrola “Ayudas a la Investigación en Energía y Medio Ambiente 2018” funded DNA sequencing Universidad Politécnica de Madrid grant RP160050022 funded the rest of the work. EI and LS-H are recipient of Becas de Colaboración del Ministerio de Educación y Formación Profesional in the years 2018/19 and 2019/20, respectively.

## Conflict of interest

The authors declare that the research was conducted in the absence of any commercial or financial relationships that could be construed as a potential conflict of interest.

## Publisher’s note

All claims expressed in this article are solely those of the authors and do not necessarily represent those of their affiliated organizations, or those of the publisher, the editors and the reviewers. Any product that may be evaluated in this article, or claim that may be made by its manufacturer, is not guaranteed or endorsed by the publisher.

## References

[ref1] Agarwal R. Burley S. K. Swaminathan S. (2012). Structural insight into mechanism and diverse substrate selection strategy of L-ribulokinase. Proteins 80, 261–268. doi: 10.1002/prot.23202, PMID: 22072612 PMC3240725

[ref2] Bandyopadhyay A. Stockel J. Min H. Sherman L. A. Pakrasi H. B. (2010). High rates of photobiological H_2_ production by a cyanobacterium under aerobic conditions. Nat. Commun. 1:139. doi: 10.1038/ncomms1139, PMID: 21266989

[ref3] Barahona E. Jimenez-Vicente E. Rubio L. M. (2016). Hydrogen overproducing nitrogenases obtained by random mutagenesis and high-throughput screening. Sci. Rep. 6:38291. doi: 10.1038/srep38291, PMID: 27910898 PMC5133592

[ref4] Barbe J. Gibert I. Llagostera M. Guerrero R. (1987). DNA repair systems in the phototrophic bacterium *Rhodobacter capsulatus*. J. Gen. Microbiol. 133, 961–966. doi: 10.1099/00221287-133-4-961, PMID: 3116168

[ref5] Bianchi L. Mannelli F. Viti C. Adessi A. De Philippis R. (2010). Hydrogen-producing purple non-sulfur bacteria isolated from the trophic lake Averno (Naples, Italy). Int. J. Hydrog. Energy 35, 12216–12223. doi: 10.1016/j.ijhydene.2010.08.038

[ref6] Boran E. Özgür E. van der Burg J. Yücel M. Gündüz U. Eroglu I. (2010). Biological hydrogen production by *Rhodobacter capsulatus* in solar tubular photo bioreactor. J. Clean. Prod. 18, S29–S35. doi: 10.1016/j.jclepro.2010.03.018

[ref7] Bulen W. A. LeComte J. R. (1966). The nitrogenase system from Azotobacter: two-enzyme requirement for N_2_ reduction, ATP-dependent H_2_ evolution, and ATP hydrolysis. Proc. Natl. Acad. Sci. U. S. A. 56, 979–986. doi: 10.1073/pnas.56.3.979, PMID: 5230193 PMC219956

[ref8] Buren S. Jimenez-Vicente E. Echavarri-Erasun C. Rubio L. M. (2020). Biosynthesis of nitrogenase cofactors. Chem. Rev. 120, 4921–4968. doi: 10.1021/acs.chemrev.9b00489, PMID: 31975585 PMC7318056

[ref9] Chai Y. H. Mohamed M. Cheng Y. W. Chin B. L. F. Yiin C. L. Yusup S.. (2021). A Review on Potential of Biohydrogen Generation through Waste Decomposition Technologies. Biomass Conv. Bioref. 2, 1–26. doi: 10.1007/s13399-021-01333-z

[ref10] Chandrasekhar K. Lee Y. J. Lee D. W. (2015). Biohydrogen production: strategies to improve process efficiency through microbial routes. Int. J. Mol. Sci. 16, 8266–8293. doi: 10.3390/ijms16048266, PMID: 25874756 PMC4425080

[ref11] Chen X. Lv Y. Liu Y. Ren R. Zhao J. (2017). The hydrogen production characteristics of mixed photoheterotrophic culture. Int. J. Hydrog. Energy 42, 4840–4847. doi: 10.1016/j.ijhydene.2016.11.155

[ref12] Colbeau A. Richaud P. Toussaint B. Caballero F. J. Elster C. Delphin C.. (1993). Organization of the genes necessary for hydrogenase expression in *Rhodobacter capsulatus*. Sequence analysis and identification of two *hyp* regulatory mutants. Mol. Microbiol. 8, 15–29. doi: 10.1111/j.1365-2958.1993.tb01199.x, PMID: 8497190

[ref13] Curatti L. Ludden P. W. Rubio L. M. (2006). NifB-dependent *in vitro* synthesis of the iron-molybdenum cofactor of nitrogenase. Proc. Natl. Acad. Sci. U. S. A. 103, 5297–5301. doi: 10.1073/pnas.0601115103, PMID: 16567617 PMC1414635

[ref14] Demtröder L. Pfänder Y. Schäkermann S. Bandow J. E. Masepohl B. (2019). Nif A is the master regulator of both nitrogenase systems in *Rhodobacter capsulatus*. Microbiology 8:e921. doi: 10.1002/mbo3.921, PMID: 31441241 PMC6925177

[ref15] Elkahlout K. E. Sagir E. Alipour S. Koku H. Gunduz U. Eroglu I.. (2019). Long-term stable hydrogen production from acetate using immobilized *Rhodobacter capsulatus* in a panel photobioreactor. Int. J. Hydrog. Energy 44, 18801–18810. doi: 10.1016/j.ijhydene.2018.10.133

[ref16] Elsen S. Dischert W. Colbeau A. Bauer C. E. (2000). Expression of uptake hydrogenase and molybdenum nitrogenase in *Rhodobacter capsulatus* is coregulated by the RegB-RegA two-component regulatory system. J. Bacteriol. 182, 2831–2837. doi: 10.1128/JB.182.10.2831-2837.2000, PMID: 10781552 PMC101992

[ref17] Feng J. Yang H. Guo L. (2018a). The photosynthetic hydrogen production performance of a newly isolated Rhodobacter capsulatus JL1 with various carbon sources. Int. J. Hydrog. Energy 43, 13860–13868. doi: 10.1016/j.ijhydene.2018.03.144

[ref18] Feng J. Yang H. Wang X. Guo L. (2018b). Enhanced hydrogen production performance of *cbbR* & *pycA* inactived *R. sphaeroides* mutant by improving the ammonium tolerance. Int. J. Hydrog. Energy 43, 18142–18150. doi: 10.1016/j.ijhydene.2018.07.196

[ref19] Fiβler J. Kohring G. W. Giffhorn F. (1995). Enhanced hydrogen production from aromatic acids by immobilized cells of *Rhodopseudomonas palustris*. Appl. Microbiol. Biotechnol. 44, 43–46. doi: 10.1007/BF00164478

[ref20] Gupta S. K. Kumari S. Reddy K. Bux F. (2013). Trends in biohydrogen production: major challenges and state-of-the-art developments. Environ. Technol. 34, 1653–1670. doi: 10.1080/09593330.2013.822022, PMID: 24350426

[ref21] Jahn A. Keuntje B. Dörffler M. Klipp W. Oelze J. (1994). Optimizing photoheterotrophic H_2_ production by Rhodobacter capsulatus upon interposon mutagenesis in the hupL gene. Appl. Microbiol. Biotechnol. 40, 687–690. doi: 10.1007/BF00173330, PMID: 7765318

[ref22] Jiménez-Llanos J. Ramírez-Carmona M. Rendón-Castrillón L. Ocampo-López C. (2020). Sustainable biohydrogen production by chlorella sp. microalgae: a review. Int. J. Hydrog. Energy 45, 8310–8328. doi: 10.1016/j.ijhydene.2020.01.059

[ref23] Joshi S. M. Inamdar S. A. Jadhav J. P. Govindwar S. P. (2013). Random UV mutagenesis approach for enhanced biodegradation of sulfonated azo dye, green HE4B. Appl. Biochem. Biotechnol. 169, 1467–1481. doi: 10.1007/s12010-012-0062-5, PMID: 23315264

[ref24] Kim E.-J. Lee M.-K. Kim M.-S. Lee J. K. (2008). Molecular hydrogen production by nitrogenase of *Rhodobacter sphaeroides* and by Fe-only hydrogenase of *Rhodospirillum rubrum*. Int. J. Hydrog. Energy 33, 1516–1521. doi: 10.1016/j.ijhydene.2007.09.044

[ref25] Klein G. Klipp W. Jahn A. Steinborn B. Oelze J. (1991). The relationship of biomass, polysaccharide and H_2_ formation in the wild-type and *nifA*/*nifB* mutants of *Rhodobacter capsulatus*. Arch. Microbiol. 155, 477–482. doi: 10.1007/Bf00244965

[ref26] Krahn E. Schneider K. Muller A. (1996). Comparative characterization of H_2_ production by the conventional Mo nitrogenase and the alternative “iron only” nitrogenase of *Rhodobacter capsulatus hup*^−^ mutants. Appl. Microbiol. Biot. 46, 285–290. doi: 10.1007/s002530050818

[ref27] Laocharoen S. Reungsang A. (2014). Isolation, characterization and optimization of photo-hydrogen production conditions by newly isolated *Rhodobacter sphaeroides* KKU-PS5. Int. J. Hydrog. Energy 39, 10870–10882. doi: 10.1016/j.ijhydene.2014.05.055

[ref28] Lee B. Choi G.-G. Choi Y.-E. Sung M. Park M. S. Yang J.-W. (2014). Enhancement of lipid productivity by ethyl methane sulfonate-mediated random mutagenesis and proteomic analysis in *Chlamydomonas reinhardtii*. Korean J. Chem. Eng. 31, 1036–1042. doi: 10.1007/s11814-014-0007-5

[ref29] Liu T. Li X. Zhou Z. (2010). Improvement of hydrogen yield by *hupR* gene knock-out and *nifA* gene overexpression in *Rhodobacter sphaeroides* 6016. Int. J. Hydrog. Energy 35, 9603–9610. doi: 10.1016/j.ijhydene.2010.06.072

[ref30] Liu B.-F. Ren N.-Q. Ding J. Xie G.-J. Guo W.-Q. (2009). The effect of Ni^2+^, Fe^2+^ and Mg^2+^ concentration on photo-hydrogen production by *Rhodopseudomonas faecalis* RLD-53. Int. J. Hydrog. Energy 34, 721–726. doi: 10.1016/j.ijhydene.2008.11.033

[ref31] Ma H. Yang H. Zheng X. Lie T. Yan W. (2021). Promoting photo-fermentative hydrogen production performance by substituting the *rnf* promoter in *Rhodobacter capsulatus*. Int. J. Hydrog. Energy 46, 3742–3752. doi: 10.1016/j.ijhydene.2020.10.270

[ref32] Masukawa H. Inoue K. Sakurai H. Wolk C. P. Hausinger R. P. (2010). Site-directed mutagenesis of the anabaena sp. strain PCC 7120 nitrogenase active site to increase photobiological hydrogen production. Appl. Environ. Microbiol. 76, 6741–6750. doi: 10.1128/AEM.01056-10, PMID: 20709836 PMC2953041

[ref33] Masukawa H. Mochimaru M. Sakurai H. (2002). Disruption of the uptake hydrogenase gene, but not of the bidirectional hydrogenase gene, leads to enhanced photobiological hydrogen production by the nitrogen-fixing cyanobacterium *anabaena* sp. PCC 7120. Appl. Microbiol. Biotechnol. 58, 618–624. doi: 10.1007/s00253-002-0934-7 11956744

[ref34] Miller J. H. (1972). Experiments in Molecular Genetics. New York, NY: Cold Spring Harbor Laboratory.

[ref35] Perin G. Bellan A. Segalla A. Meneghesso A. Alboresi A. Morosinotto T. (2015). Generation of random mutants to improve light-use efficiency of *Nannochloropsis gaditana* cultures for biofuel production. Biotechnol. Biofuels 8:161. doi: 10.1186/s13068-015-0337-5, PMID: 26413160 PMC4583171

[ref36] Plovins A. Alvarez A. M. Ibañez M. Molina M. Nombela C. (1994). Use of fluorescein-di-beta-D-galactopyranoside (FDG) and C12-FDG as substrates for beta-galactosidase detection by flow cytometry in animal, bacterial, and yeast cells. Appl. Microbiol. Biotechnol. 60, 4638–4641. doi: 10.1128/aem.60.12.4638-4641.1994, PMID: 7811104 PMC202038

[ref37] Rey F. E. Heiniger E. K. Harwood C. S. (2007). Redirection of metabolism for biological hydrogen production. Appl. Environ. Microbiol. 73, 1665–1671. doi: 10.1128/AEM.02565-06, PMID: 17220249 PMC1828789

[ref38] Schindel H. S. Bauer C. E. (2016). The RegA regulon exhibits variability in response to altered growth conditions and differs markedly between *Rhodobacter* species. Microb. Genom. 2:e000081. doi: 10.1099/mgen.0.000081, PMID: 28348828 PMC5359404

[ref39] Schneider K. Gollan U. Dröttboom M. Selsemeier-Voigt S. Müller A. (1997). Comparative biochemical characterization of the iron-only nitrogenase and the molybdenum nitrogenase from *Rhodobacter capsulatus*. Eur. J. Biochem. 244, 789–800. doi: 10.1111/j.1432-1033.1997.t01-1-00789.x, PMID: 9108249

[ref40] Schneider K. Müller A. Schramm U. Klipp W. (1991). Demonstration of a molybdenum- and vanadium-independent nitrogenase in a *nifHDK*-deletion mutant of *Rhodobacter capsulatus*. Eur. J. Biochem. 195, 653–661. doi: 10.1111/j.1432-1033.1991.tb15750.x, PMID: 1999188

[ref41] Schubert K. R. Evans H. J. (1976). Hydrogen evolution: A major factor affecting the efficiency of nitrogen fixation in nodulated symbionts. Proc. Natl. Acad. Sci. U. S. A. 73, 1207–1211. doi: 10.1073/pnas.73.4.1207, PMID: 16592307 PMC430231

[ref42] Scolnik P. A. Haselkorn R. (1984). Activation of extra copies of genes coding for nitrogenase in *Rhodopseudomonas capsulata*. Nature 307, 289–292. doi: 10.1038/307289a0, PMID: 6582352

[ref43] Seefeldt L. C. Yang Z. Y. Lukoyanov D. A. Harris D. F. Dean D. R. Raugei S.. (2020). Reduction of substrates by nitrogenases. Chem. Rev. 120, 5082–5106. doi: 10.1021/acs.chemrev.9b00556, PMID: 32176472 PMC7703680

[ref44] Simpson F. B. Burris R. H. (1984). A nitrogen pressure of 50 atmospheres does not prevent evolution of hydrogen by nitrogenase. Science 224, 1095–1097. doi: 10.1126/science.6585956 6585956

[ref45] Skizim N. J. Ananyev G. M. Krishnan A. Dismukes G. C. (2012). Metabolic pathways for photobiological hydrogen production by nitrogenase- and hydrogenase-containing unicellular cyanobacteria Cyanothece. J. Biol. Chem. 287, 2777–2786. doi: 10.1074/jbc.M111.302125, PMID: 22128188 PMC3268435

[ref46] Strnad H. Lapidus A. Paces J. Ulbrich P. Vlcek C. Paces V.. (2010). Complete genome sequence of the photosynthetic purple nonsulfur bacterium *Rhodobacter capsulatus* SB 1003. J. Bacteriol. 192, 3545–3546. doi: 10.1128/JB.00366-10, PMID: 20418398 PMC2897665

[ref47] Vignais P. M. Billoud B. (2007). Occurrence, classification, and biological function of hydrogenases: an overview. Chem. Rev. 107, 4206–4272. doi: 10.1021/cr050196r, PMID: 17927159

[ref48] Vignais P. M. Elsen S. Colbeau A. (2005). Transcriptional regulation of the uptake [NiFe] hydrogenase genes in *Rhodobacter capsulatus*. Biochem. Soc. Trans. 33, 28–32. doi: 10.1042/BST0330028, PMID: 15667256

[ref49] Wang D. Zhang Y. Welch E. Li J. Roberts G. P. (2010). Elimination of Rubisco alters the regulation of nitrogenase activity and increases hydrogen production in *Rhodospirillum rubrum*. Int. J. Hydrog. Energy 35, 7377–7385. doi: 10.1016/j.ijhydene.2010.04.183, PMID: 20652089 PMC2905822

[ref50] Weaver P. F. Wall J. D. Gest H. (1975). Characterization of *Rhodopseudomonas capsulata*. Arch. Microbiol. 105, 207–216. doi: 10.1007/BF00447139 1103769

[ref51] Zhang Y. Yang H. Feng J. Guo L. (2016). Overexpressing F0/F1 operon of ATPase in *Rhodobacter sphaeroides* enhanced its photo-fermentative hydrogen production. Int. J. Hydrog. Energy 41, 6743–6751. doi: 10.1016/j.ijhydene.2016.03.061

[ref52] Zheng Y. Harwood C. S. (2019). Influence of energy and electron availability on *in vivo* methane and hydrogen production by a variant molybdenum nitrogenase. Appl. Environ. Microbiol. 85, e02671–e02618. doi: 10.1128/aem.02671-18 30824440 PMC6495768

