# Supplementary material for: A directed genome evolution method to enhance hydrogen production in Rhodobacter capsulatus
Source: Front Microbiol. 2022 Aug 24;13:991123. doi: 10.3389/fmicb.2022.991123 (PMC9449697; doi:10.3389/fmicb.2022.991123)
Supplement: Supplementary file 1 [file Data_Sheet_1.PDF]

Supplementary Material

1 Supplementary Figures

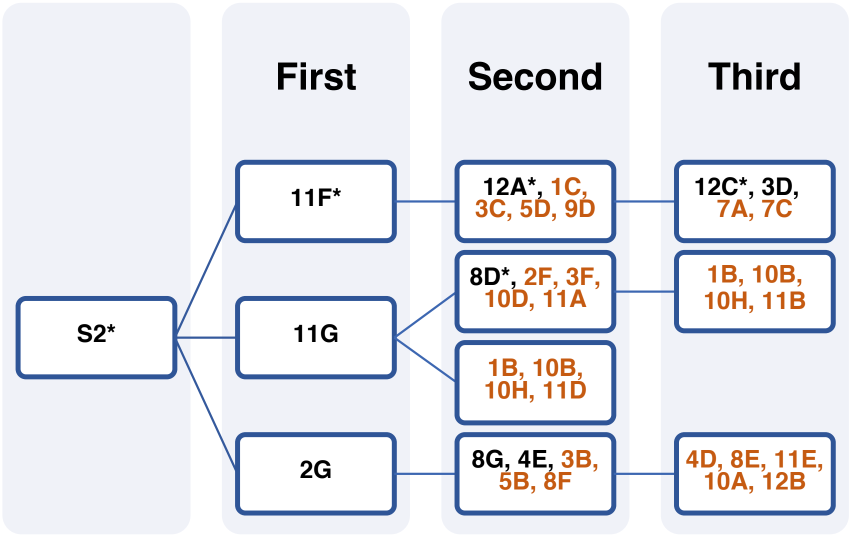

Supplementary Figure S1. Genealogy of *R. capsulatus* strains generated by UV-mutagenesis. The parental strain S2 is shown in the first column. Results of each round of UV mutagenesis are shown in the next three columns. Asterisks indicate strains which genomes have been sequenced. Strains for which H_2 production was determined (shown in Figure S2) but were not investigated further are in brown.

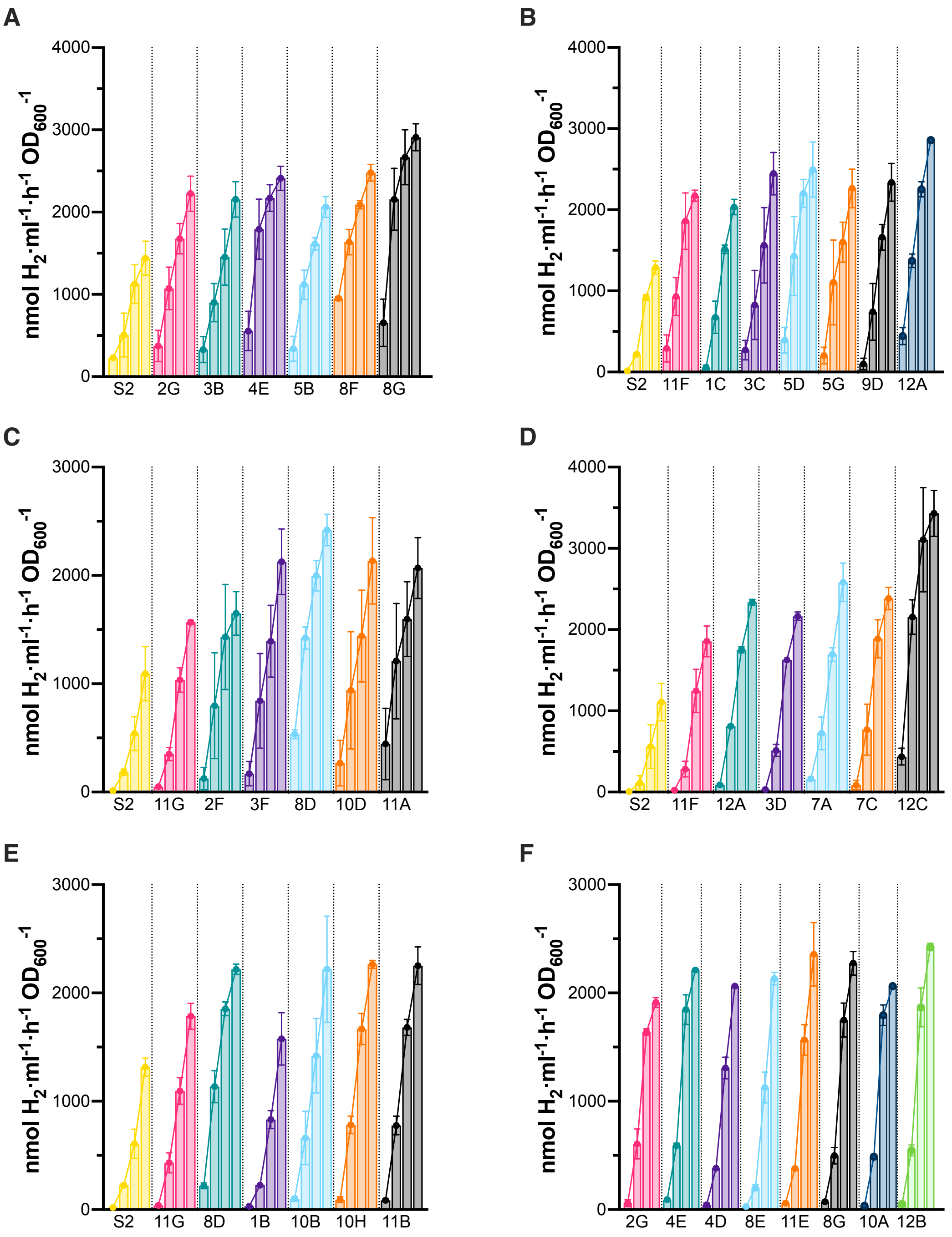

Supplementary Figure S2. Time course of H_2 production in batch cultures of strains selected after UV mutagenesis. See Figure S1 for strain genealogy. **(A)** 2G and its derivatives. **(B)** 11F and its derivatives. **(C)** 11G and its derivatives. **(D)** 12A and its derivatives. **(E)** 8D and its derivatives. **(F)** 8G, 4E and their derivatives. The four bars of each strain represent activities at 13, 16, 19, and 22 hours after the start of nitrogenase derepression in RCV_0 medium. Data represent the mean ± SD of at least 2 biological replicates with 2 technical replicates each.

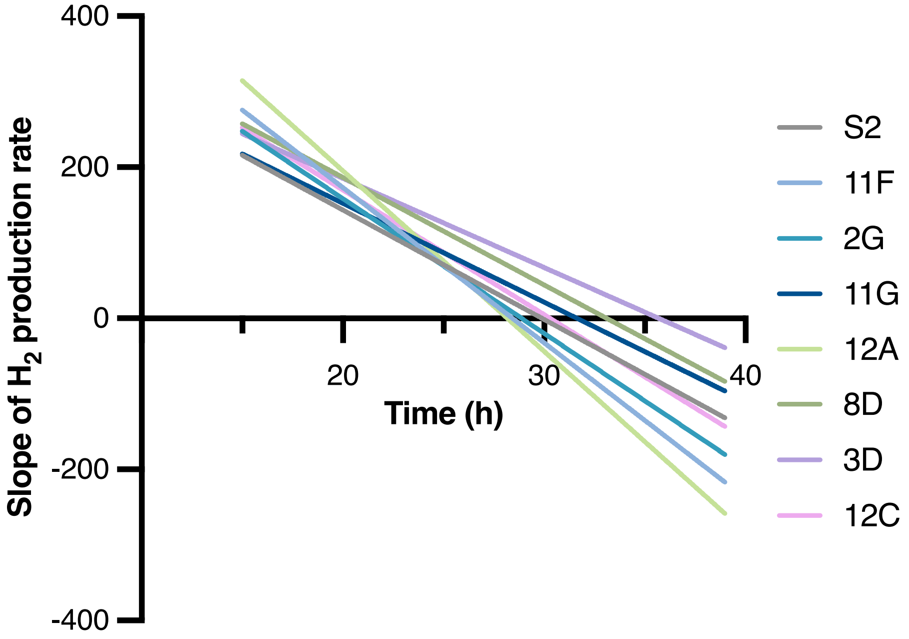

Supplementary Figure S3. Deceleration of H_2 production rates over time. The graph shows the first derivative of H_2 production rates of mutant strains from 15 to 39 hours after nitrogenase derepression. Note negative slopes (decreasing rates) appearing between 28 and 39 hours.

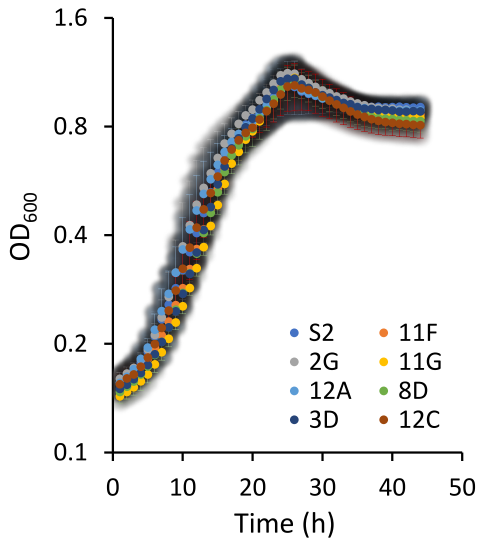

Supplementary Figure S4. Growth of S2 and selected derivative strains in batch cultures. Data represent means (dots) and SD (shade areas) for n = 3 biological replicates.

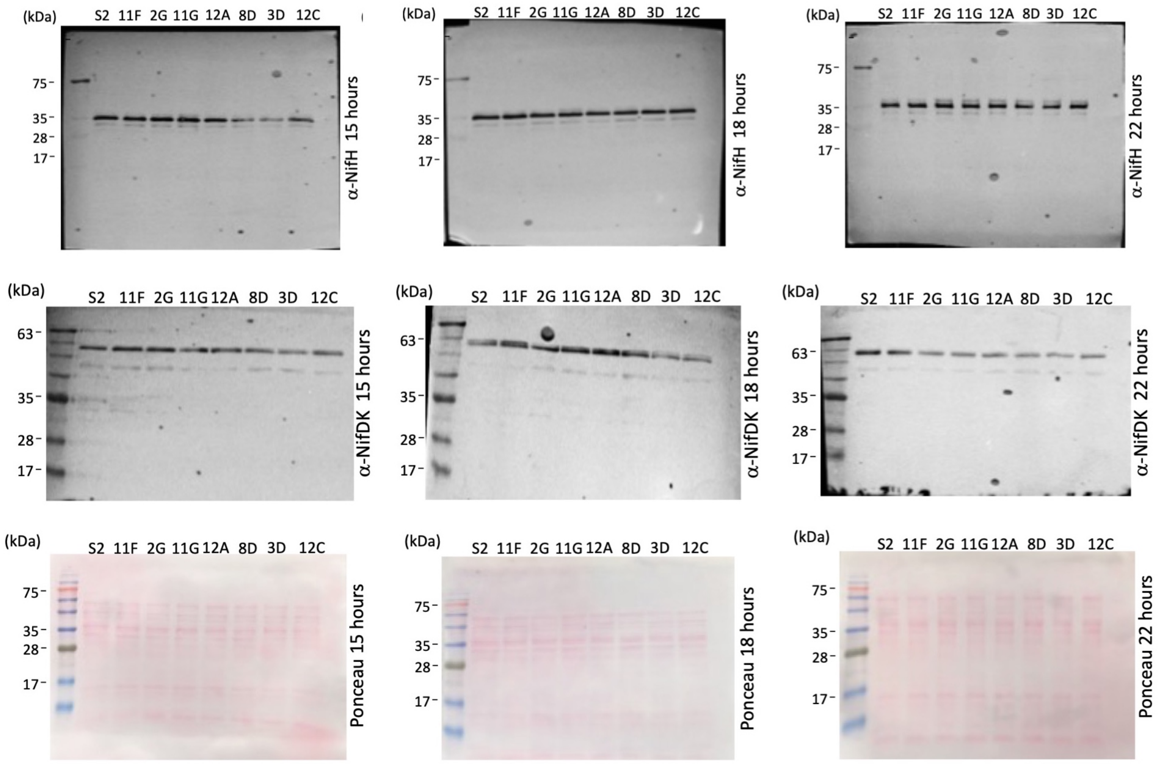

Supplementary Figure S5. Uncropped immunoblots and membranes shown in Figure 3C.

2 Supplementary Tables

Supplementary Table S1. Strains used in this work.

R. capsulatus strains	Characteristics	Source/ Reference
R. capsulatus SB1003 (WT)	Spontaneous Rif^R mutant derived from B10 wild-type strain	(Cullen et al., 1997)
S1	WT harboring a chromosomal translation fusion P*hupA*::*lacZ* (Rif^R, Km^R)	(Barahona et al., 2016)
S2	Δ*hupAB* harboring a chromosomal translation fusion P*hupA*::*lacZ* (Rif^R, Km^R)	(Barahona et al., 2016)
11F	S2-derivative strain from the first round of UV mutagenesis (Rif^R, Km^R)	This work
2G	S2-derivative strain from the first round of UV mutagenesis (Rif^R, Km^R)	This work
11G	S2-derivative strain from the first round of UV mutagenesis (Rif^R, Km^R)	This work
12A, 1C, 3C, 5D, and 9D	11F-derivative strains from the second round of UV mutagenesis (Rif^R, Km^R)	This work
8D, 2F, 3F, 10D, and 11A	11G-derivative strains from the second round of UV mutagenesis (Rif^R, Km^R)	This work
8G, 4E, 3B, 5B, and 8F	2G-derivative strains from the second round of UV mutagenesis (Rif^R, Km^R)	This work
10A and 12B	8G-derivative strains from the second round of UV mutagenesis (Rif^R, Km^R)	This work
4D, 8E, and 11E	4E-derivative strains from the second round of UV mutagenesis (Rif^R, Km^R)	This work
3D, 12C, 7A, and 7C	12A-derivative strains from the third round of UV mutagenesis (Rif^R, Km^R)	This work
1B, 10B, 10H, and 11B	8D -derivative strains from the third round of UV mutagenesis (Rif^R, Km^R)	This work

Supplementary Table S2. Detailed information about *R. capsulatus* mutant strains (Excel dataset).

3 Supplementary References

Barahona, E., Jimenez-Vicente, E., and Rubio, L.M. (2016). Hydrogen overproducing nitrogenases obtained by random mutagenesis and high-throughput screening. *Sci. Rep.* 6, 38291. doi: 10.1038/srep38291.

Cullen, P.J., Kaufman, C.K., Bowman, W.C., and Kranz, R.G. (1997). Characterization of the *Rhodobacter capsulatus* housekeeping RNA polymerase. *In vitro* transcription of photosynthesis and other genes. *J. Biol. Chem.* 272, 27266-27273. doi: 10.1074/jbc.272.43.27266.
